# Waveform characteristics in thoracic paravertebral space: a prospective observational study

**DOI:** 10.12688/f1000research.139904.1

**Published:** 2024-03-01

**Authors:** Amorn Vijitpavan, Sivaporn Termpornlert, Pattika Subsoontorn, Lalinthip Vareesunthorn

**Affiliations:** 1 Department of Anesthesiology, Faculty of Medicine Ramathibodi Hospital, Mahidol University, Ratchathewi, Bangkok, 10400, Thailand

**Keywords:** Thoracic paravertebral block, Respiratory waveform, Pressure value, Thoracic paravertebral space, Observational study, Ultrasound-guided

## Abstract

**Background:**

With increased use of thoracic paravertebral block (TPVB) in thoracic surgery, many faced the challenge of locating the thoracic paravertebral space (TPVS) ultrasonographically. This observational study aimed to investigate the waveform characteristics and pressure value within the TPVS in anaesthetized patients with controlled ventilation.

**Methods:**

50 patients scheduled for elective lung surgery were enrolled. After conduction of anesthesia, all patients underwent TPVB at T4/5 and T6/7 using transverse, in-plane ultrasound guidance. A pressure transducer system with a desktop monitor was connected to the needle hub to measure pressure values and waveform characteristics in three locations: the paraspinal muscles, immediately behind the superior costotransverse ligament, and within the TPVS. Next, 15 mL of 0.33% bupivacaine was injected into each desired TPVS. After completion of the surgery, the extent of dermatomal blockade and the pain score was assessed in all patients.

**Results:**

98 typical regular respiratory waveforms with a mean pressure of ≤ 25 mmHg were detected in the TPVS of 50 patients. The sensitivity of the combined ultrasound and pressure waveform measurement technique to identify the TPVS was 95.45% (95% confidence interval, 84.527–99.445). Nontypical respiratory waveforms were present in two patients. Factors interfering with the TPVS waveform characteristics were previous thoracic surgery and chronic pleural inflammation.

**Conclusion:**

The TPVS had low pressure and showed a smooth, regular waveform pattern corresponding to respiration.

## Introduction

Thoracic paravertebral block (TPVB) has become an increasingly popular technique for pain control after thoracic surgery through various approaches such as the anatomical-based technique. ^1^ However, this technique has a failure rate of up to 10% and is associated with several potential adverse events: hypotension (4.6%), vascular puncture (3.8%), pleural puncture (1.1%), pneumothorax (0.5%), hematoma (1.9%), and epidural or spinal spread (1.1%) ^2^ Identifying the thoracic paravertebral space (TPVS) is crucial in achieving success and preventing adverse events. Numerous methods have been proposed to locate the TPVS, such as the loss-of resistance technique, ^3^ nerve stimulation, ^4^^–^^7^ pressure measurement, ^8^^–^^10^ and an acoustic signal. ^11^ Ultrasound guidance is another approach for TPVB and was first introduced by Hara *et al.* ^12^ for breast surgery. The evidence to date demonstrates that ultrasound-guided TPVB (UG-TPVB) is safe to perform in sedated and ventilated patients. ^13^ Most anesthesiologists currently use ultrasound to locate the TPVS and needle path. Patnaik *et al.* ^14^ reported that UG-TPVB resulted in a more successful block than the anatomical landmark technique (94% and 72%, respectively), although the complication rates were comparable (13.8% and 22.2%, respectively). Several approaches can be used for UG-TPVB, such as a parasagittal or transverse probe orientation and an in-plane or out-of-plane technique for needle visualization. ^15^^,^^16^ However, the optimal UG-TPVB method remains unclear. ^15^

Practically, observation of the needle tip by ultrasound while simultaneously advancing the needle to the target area surrounded by the bone is challenging. The TPVS is a narrow channel adjacent to the lung and spinal canal, and the needle tip may be misplaced into a non-target area, causing block failure and complications. ^17^ As the TPVS lies adjacent to the pleural space, we speculated that the characteristics of the respiratory waveform could be detected from the needle tip once it was located within the TPVS. Thus, this study aimed to observe waveform patterns and pressure values in the TPVS when performing UG-TPVB after the induction of general anesthesia with controlled ventilation.

## Methods

This prospective observational study was provided by the Ethics Committee of the Ramathibodi Hospital, Mahidol University, Bangkok, Thailand on 19 ^th^ May 2020 (reference no. MURA2020/860). The protocol for this study was registered in the protocols.io repository ( https://www.protocols.io/view/waveform-characteristics-in-thoracic-paravertebral-14egn3mxpl5d/v1).

A total of 50 patients scheduled for elective lung surgery (open thoracotomy and video-assisted thoracoscopic surgery) were recruited during 9 ^th^ February 2021 to 17 ^th^ May 2021. Anesthesiology residents or fellows at Ramathibodi Hospital obtained preoperative information and provided informed consent. The inclusion criteria were an age of 18 to 80 years and an American Society of Anesthesiologists (ASA) physical status of I to III. The ASA physical status was defined as the following: ASA I is a normal healthy patient, ASA II is a patient with mild systemic disease, ASA III is a patient with severe systemic disease, ASA IV is a patient with severe systemic disease that is a constant threat to life, ASA V is a moribund patient who is not expected to survive without the operation. ^18^

The exclusion criteria were no provision of informed consent, refusal to receive UG-TPVB, a body mass index of >35 kg/m ^2^, significant thoracic kyphoscoliosis, coagulopathy (platelet count of <100,000 per mL or international normalized ratio of >1.4), allergies or contraindications to medications used in the study protocol, and refusal to participate or withdrawal of consent at any stage of the study.

All enrolled patients received standard protocol for elective lung surgery. They were fasted for at least eight hours before surgery. Standard ASA monitoring was performed throughout the surgery. Anesthesia was induced using propofol (2.0–2.5 mg/kg), fentanyl (1–2 mcg/kg), and cisatracurium (0.15–0.2 mg/kg) intravenously. Patients were intubated with left-sided double-lumen tubes. The anesthetized patient was placed in the lateral decubitus position with the operative site up for UG-TPVB. During UG-TPVB, both lungs were ventilated with continuous positive pressure ventilation (pressure-controlled mode with inspiratory pressure of 20 cmH _2_O, inspiratory time of 1 second, respiratory rate of 12 breaths per minute, and positive end-expiratory pressure of 5 cmH _2_O). Under sterile conditions, UG-TPVB was performed with a high-frequency linear transducer (12-MHz 9L-RS probe, GE Vivid IQ ultrasound machine; GE Healthcare, Chicago, IL, USA) by experienced operators using the transverse in-plane technique at the fourth and the fifth thoracic vertebrae (T4/5) and the sixth and the seventh thoracic vertebrae (T6/7) of the TPVS. The ultrasound probe was covered with a sterile plastic sleeve and placed on the back of the shoulder area. The operator identified the first rib and then counted downward until the fourth rib was reached. The probe was then moved inward to locate the fourth transverse process. The ultrasound probe was then dragged downward to locate the fifth transverse process and identify the location of the TPVS between T4 and T5, labelling the site with an indelible pen. The ultrasound probe was moved further downward to locate the sixth and seventh transverse processes and the TPVS was marked between T6 and T7.

**Figure 1.**
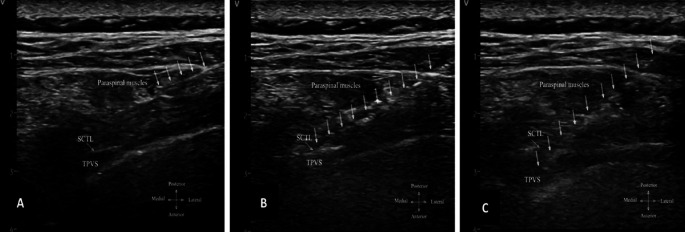
Ultrasound image of thoracic paravertebral block and needle tip position during pressure measurement. (A) Paraspinal muscle. (B) Immediately posterior to SCTL. (C) In TPVS. Abbreviation: SCTL, superior costotransverse ligament; TPVS, thoracic paravertebral space; dotted arrow represents the needle path.

An echogenic needle (SonoTAP; Pajunk GmbH Medizintechnologie, Geisingen, Germany) was connected to a pressure transducer system (TruWave PX260; Edwards Lifesciences, Irvine, CA, USA) *via* a three-way stopcock (Discofix 3SC; B. Braun, Melsungen, Germany) and a 36-inch noncompliant pressure tubing (Edwards Lifesciences). The pressure transducer was connected to a desktop monitor (IntelliVue MP70; Philips, Amsterdam, Netherlands) and levelled at the spinous process. The needle was then inserted at the skin approximately 3 cm from the midline and advanced laterally to medially under in-plane ultrasound visualization. We observed the mean pressure and waveform characteristics when the needle tip was in three locations ( Figure 1).

First, the tip of the needle was identified in the paraspinal muscles. Second, the needle tip was advanced immediately posterior to the superior costotransverse ligament (SCTL) and confirmed by a 1.0-mL normal saline injection. Third, the needle tip was located in the TPVS and confirmed by a 0.5- to 3.0-mL saline injection, which widened the TPVS and caused anterior displacement of the pleura.

The operator then slowly injected 15 mL 0.33% bupivacaine into each desired TPVS. The collected waveforms were classified into three patterns (A, B, and C) according to our pilot study and based on previous trials. ^8^^–^^11^ The “A” waveform or typical waveform was defined as a smooth and regular sine wave resembling the respiratory pattern with a mean value of ≤ 25 mmHg; the “B” waveform was defined as an irregular coarse, wavy line; and the “C” waveform was defined as a tense, straight line. B and C waveforms were referred to as atypical waveform.

The attending anesthesiologists maintained anesthesia to achieve adequate anesthetic depth using 1:1 of air: oxygen, 2% sevoflurane, fentanyl, and cisatracurium. After completion of surgery, all patients were extubated and transferred to the postanesthetic care unit.

The objectives of this study encompassed the observation of thoracic paravertebral waveforms and pressure values in TPVS, as well as an assessment of the efficacy of UG-TPVB. This evaluation was accomplished by gauging the subjects’ Numeric Rating Scale (NRS) scores and the extent of dermatomal blockade, determined by pinprick sensation, once they regained full consciousness. An unsuccessful block was characterized as the inability to confirm more than three levels of dermatomal blockade, as per Eason and Wyatt’s criteria, or an NRS score at rest ≥3. ^3^

Continuous variables were presented as mean ± standard deviation or median ± range as appropriate after Shapiro-Wilk and Shapiro-Francia tests for normality. Categorical variables were presented as numbers and percentages. The quantile regression was applied to test the median difference between injection site, multiple comparison, thoracotomy group, and the difference between the median pressure values of T4/5 and T6/7 to estimate the sensitivity and positive predictive values. All statistical analysis was calculated by STATA 17 (StataCorp. 2021. Stata Statistical Software: Release 17. College Station, TX: StataCorp LLC; RRID: SCR_012763). The significance was set at p-value of < 0.05.

## Results

50 patients were recruited between February 2021 and May 2021. Their demographic and perioperative data are summarized in Table 1.

**Table 1.** Demographic and perioperative data.

Demographic and perioperative data	Statistics
Age, years, mean±SD	60.8±13.0
Sex, n (%)	
Male	16 (32.0)
Female	34 (68.0)
Body mass index, kg/m ^2^, mean±SD	24.2±3.9
Operative side, n (%)	
Right	32 (64.0)
Left	18 (36.0)
Operation type	
VATS, n (%)	37 (74.0)
Lobectomy	27 (78.0)
Segmentectomy	5 (13.5)
Wedge resection	3 (8.1)
Blebectomy	2 (5.4)
Open thoracotomy, n (%)	13 (26.0)
Lobectomy	9 (69.2)
Wedge resection	1 (7.7)
Decortication	2 (15.4)
Blebectomy	1 (7.7)
Previous ipsilateral thoracic surgery, n (%)	4 (8.0)
Mean inspiratory pressure during two-lung ventilation, mmHg, mean±SD	14.0±1.54
Dynamic lung compliance, ml/cm H _2_O, mean±SD	42.7±15.5
Operation time, minutes, mean±SD	275.7±68.5
Total intraoperative fentanyl, mcg, mean±SD	109.5±45.1
Failed block, n (%)	6 (12.0)
Dermatomal blocks, levels, mean±SD	4.9±1.5

Abbreviation: VATS, video-assisted thoracoscopic surgery.

Continuous variables were presented as mean ± standard deviation (range), and categorical variables were presented as number (%).

**Figure 2.**
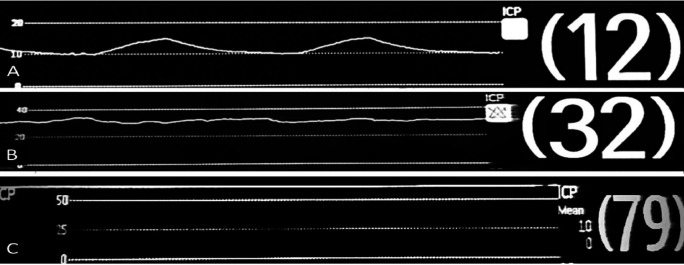
Waveform characteristics (A) needle tip position within TPVS and (B, C) needle tip position outside TPVS.

All patients were extubated and assessed for dermatomal sensory blockade and NRS scores before discharge from the post-anaesthetic care unit. Compared with the pressure values at each position of the needle at both levels, the pressures in the TPVS were significantly different from the pressures from the paraspinal muscle and SCTL as shown in Table 2. The median pressure value in the TPVS of T4/5 was 17.0 (3.0, 47.0) mmHg, and that of T6/7 was 15.5 (8.0, 26.0) mmHg. There was significant difference in the pressure value between T4/5 and T6/7 (p < 0.001). The waveform characteristics were presented in Table 3 and Figure 2. The number B and C waveform were counted as an atypical waveform group; the intra-muscle and SCTL layers were combined together as non TPVS. When compared with the UG- TPVB, the sensitivity, specificity, positive and negative predictive value (PPV, NPV) and receiver operating characteristic (ROC) area of typical waveform to locate the TPVS were 98.0 (93.0, 99.8), 99.5 (97.2, 100.0), 99.0 (94.5, 100.0), 99.0 (96.5, 99.9), 99.8 (97.3, 100.0), respectively ( Table 4).

**Table 2.** Mean pressure values.

Injection site	T 4/5 median (range)	T 6/7 median (range)	P-value
^a^Intramuscular pressure, mmHg	44.0 (5.0, 95.0)	45.5 (14.0, 85.0)	0.154
^b^SCTL pressure, mmHg	34.5 (20.0, 81.0)	40.0 (23.0, 95.0)	<0.001 *
^c^TPVS Pressure, mmHg	17.0 (3.0, 47.0)	15.5 (8.0, 26.0)	<0.001 *
Respiratory waveform present, %	94.0	100.0	
Overall p-value	<0.001 *	<0.001 *	
Multiple comparison			
a vs. b	0.001 *	0.063	
a vs. c	<0.001 *	<0.001 *	
b vs. c	<0.001 *	<0.001 *	
Redo thoracotomy (n=4)			
Intramuscular pressure, mmHg	59.5 (27.0, 95.0)	47.0 (37.0, 68.0)	
SCTL pressure, mmHg	49.0 (43.0, 62.0)	44.5 (35.0, 95.0)	
TPVS Pressure, mmHg	20.5 (18.0, 37.0)	18.0 (4.0, 26.0)	
Respiratory waveform present, %	100.0	100.0	
Not redo thoracotomy (n=46)			
Intramuscular pressure, mmHg	43.0 (5.0, 86.0)	45.0 (14.0, 85.0)	
SCTL pressure, mmHg	34.0 (20.0, 81.0)	38.0 (23.0, 80.0)	
TPVS Pressure, mmHg	16.0 (3.0, 47.0)	15.5 (8.0, 25.0)	
Respiratory waveform present, %	93.5	100.0	

Abbreviations: SCTL, superior costotransverse ligament; TPVS, thoracic paravertebral space.

Data are presented as median (range).

* P<0.05 (statistically significant).

**Table 3.** Waveform characteristics.

Waveform characteristics (n=100)	Intramuscular	SCTL	TPVS
**A** Regular sine respiratory waveform with mean pressure of ≤25 mmHg	0	1	98
**B** Irregular course wavy line with mean pressure of ≤40 mmHg	33	51	1
**C** Tense straight line with mean pressure of >40 mmHg	67	48	1

Abbreviations: SCTL, superior costotransverse ligament; TPVS, thoracic paravertebral space.

Data were presented as number (n).

Sensitivity:          98.0 (93.0, 99.8)   Specificity:        99.5 (97.2, 100.0).

ROC area:         99.8 (97.3, 100.0).

Positive predictive value:   99.0 (94.5, 100.0).  Negative predictive value: 99.0 (96.5, 99.9).

**Table 4.** Sensitivity and specificity of waveform visibility test.

Typical respiratory waveform	Successful block (n)	Unsuccessful block (n)
Present	42	6
Absent	2	0
		
Sensitivity:	95.45% (95% confidence interval, 84.52%–99.44%)	
Positive predictive value:	83.36% (95% confidence interval, 82.45%–84.24%)	

Data were presented as number (n).

50 patients had 98 typical respiratory waveforms within the TPVS. The pressure values of the TPVS at T4/5 and T6/7 of 5 patients with redo-thoracotomy were 20.5 (18.0, 37.0) mmHg and 18.0 (4.0, 26.0) mmHg respectively with no significant differences between both levels. Two successful UG-TPVB showed an atypical waveform with a mean pressure of >25 mmHg. The sensitivity of the pressure value and waveform characteristics to identify TPVS was 95.45% (95% confidence interval, 84.527–99.445) when successful dermatomal blockade was used as reference ( Table 4).

Nevertheless, six patients who underwent unsuccessful block exhibited typical respiratory waveforms in the TPVS. The specificity of the study was limited because none of the patients who underwent unsuccessful block showed an absent TPVS waveform.

The overall mean NRS scores for patients at rest was 2.52±2.32 (0–8) and on movement was 4.06±2.40 (0–10). Additionally, patients with redo thoracotomy and without redo thoracotomy at rest and on movement scored similarly with no significant difference (at rest: 4.6±2.97 (0-8) vs 2.29±2.16 (0-7) respectively, p = 0.164) (on movement: 6.4±2.61 vs 3.8±2.26 respectively, p = 0.164).

## Discussion

There was a significant difference in the waveform characteristics and pressure values in the TPVS and the surrounding outer structures. As the needle passed through TPVS, the pressure monitoring showed that pressure values of T4/5 and T6/7 dropped to the lowest level which parallel to a sudden transition from a tense straight line (C -waveform) or irregular wave (B-waveform) to an “A” waveform that resembled to the respiratory waveforms corresponding to the ventilator’s setting. The apparent differences in the pressure values and waveform patterns in each position reflected the dynamic effect of the pressure transmitted from the thoracic cavity to the adjacent area such as the TVPS. The study showed that 96% of the subjects had similar respiratory-like waveform pattern in the TPVS. Thus, these differences in each position can be utilized to locate the TPVS during TPVB. The sensitivity, specificity and PPV of waveform to identify TPVS were 98% and 99.5%, 99%, respectively, when ultrasound was used as reference, Similarly, the sensitivity and PPV were 95.5%, 83.4%, respectively when successful dermatomal blockade was used as reference. Therefore, both pressure values and waveform characteristics can be applied as an adjunct to locate the TPVS.

On the contrary, Richardson *et al.* used a sudden pressure drop to identify the TPVS. ^8^ The average pressure in the TPVS was 7.6 mmHg for mean expiratory pressure and 3.3 mmHg for inspiratory pressure, which were lower than the mean pressure derived from our study. As in our study, Okitsu *et al.* reported the pressure value (<30 mmHg) in the TPVS after induction of general anesthesia with the patients in the decubitus position which was close to our study (25 mmHg). ^9^ The lower pressure value reported by Richardson *et al.* could be due to their subjects were spontaneously breathing, whereas in our study and Okitsu *et al.* performed in subjects who underwent positive pressure ventilation. ^8^^,^^9^

Prior insults to the pleura might influence the pressure and wave configuration in the TPVS. In this study, there were four patients with a history of ipsilateral thoracotomy, and two were diagnosed with empyema thoracis. However, these patients had a fairly high pressure in TPVS. They also had a relatively high NRS score with borderline statistical significance [at rest: 4.6±2.96 (p=0.07), during movement: 6.4±2.61 (p=0.05)] when compared with patients who had an innocent pleural lining. Although there were some discrepancies between the statistical significances for the pressure value at different locations of the two vertebral levels, the sample size of only four patients with redo-thoracotomy was insufficient to represent the pressure values at each location and its effect on pain control.

Inevitable adhesion formation and inflammation post-thoracotomy tends to alter or obliterate the TPVS. ^19^ Cheema *et al.* reported that extrapleural adhesions and scar tissue after the previous thoracotomy may be technically more challenging. ^20^ The disrupted pleura cannot contain the infused local anaesthetic agent, diminishing the analgesic efficacy of TPVB. A comparative study on the importance of pleural integrity for safe TPVB by Komatsu *et al.* showed that patients with a previous pleural tear required significantly more rescue medications on the first postoperative day. ^21^

The waveform characteristics in patients who underwent redo-thoracotomy or empyema were almost identical to those in patients with normal pleura. Eleventh of the twelve waves from six patients had a typical respiratory waveform (“A” waveform) in the TPVS. The rest of the patients in redo-thoracotomy group had a regular respiratory-like waveform, but the mean pressure was >25 mmHg. Therefore, this study confirmed that the characteristics of the respiratory waveform can be used to identify TPVS, even, in patients with pleural disorder.

One patient with a history of redo-thoracotomy showed a pulsatile waveform synchronized with arterial pulsations. This event occurred when the needle tip was obscurely located immediately deep to the SCTL during ultrasound scanning. Similar to a patient with empyema thoracis, a pulsatile waveform also appeared as the needle tip moved into the TPVS. None of the patients showed signs of widening or anterior displacement of the pleura after testing with saline injection and arterial puncture was ruled out by blood aspiration. Compared with the specific pulsatile waveforms in the epidural space produced by previous analysis studies, we deduced that the needle tip may have been in the epidural space and the respiratory waveform was displayed after repositioning of the needle path. ^22^^,^^23^ Therefore, patients with a distorted pleural lining from previous surgery may have TPVS waveform alterations, and TPVB should be performed with caution.

Nontypical respiratory waveforms within the TPVS were found in two patients with a successful block. The first patient showed a tense, straight waveform with a mean pressure of 47 mmHg, which might be explained by the needle tip contacting a bony part ( *e.g.,* transverse process or vertebral body) or the pleura during pressure measurement, resulting in falsely high pressure without a typical sine wave. Costache *et al.* postulated that blockade of the thoracic nerve roots in the TPVS can be achieved through several injection points outside the TPVS as paravertebral block variants. ^24^ The second patient with a history of thoracic surgery demonstrated a respiratory waveform with a mean pressure of 37 mmHg, which was defined as an atypical waveform. This pattern might be explained by the scar of redo-thoracic surgery on the same side, which could have affected pleural integrity and compliance in the TPVS.

Six patients underwent unsuccessful blocks despite the needle tip being ultrasonographically visible in the TPVS along with widening of the TPVS and anterior displacement of the pleura being observed after confirmation by saline injection. Furthermore, a typical respiratory waveform was observed in all these patients. This unfavourable result might be explained by the uncontrollable variations in the spread of local anaesthetic. Previous cadaveric studies demonstrated direct communication between the TPVS and the intercostal space. Cowie *et al.* performed UG-TPVB with contrast injection and found a greater spread of contrast in the intercostal space than in the TPVS. ^25^ Naja *et al.* used nerve stimulator and roentgenogram data to show four main types of injectate spreading patterns in TPVB: pure longitudinal (TPVS), longitudinal with intercostal (TPVS with intercostal), intercostal, and cloud-like spread around the injection sites. In addition, isolated paravertebral contrast was found in only 30% of patients. ^26^ Termpornlert *et al.* also found that the spreading of dye through a paravertebral catheter showed considerable differences in patterns. ^27^ Several factors can impact the distribution of local anaesthetic agents in the TPVS, such as the compliance in the space, pressure and injection speed, injection volume, viscosity of the local anaesthetic agent, and size of the patient. ^28^

To improve block quality, Choi *et al.* and Li *et al.* studied combined UG-TPVB and pressure measurement for TPVB and found a shorter procedure time, higher success rate, and superior analgesia compared with UG-TPVB alone. ^10^^,^^29^ The results of our study can also be applied to TPVB. A regular respiratory waveform pattern can be used as an adjunct to identify the TPVS and could be more effective than the pressure value alone.

### Limitations

The pressure and waveform pattern in the TPVS obtained in this study were derived from observing patients undergoing positive pressure ventilation, which may differ from those in individuals breathing spontaneously.

Dermatomal sensory block and NRS score assessment were performed postoperatively; analgesia might have passed its peak effect, resulting in dermatomal regression.

Each patient received UG-TPVB at two levels with a high volume of local anesthetic agent, as a routine practiced by our team; the spread might have overlapped, leading to potential misinterpretation of the blockade’s effectiveness at each level. This study was prospective observational; therefore, a randomized controlled trial is necessary to elucidate the benefits of analysing the waveform in the TPVS.

## Conclusions

The wave in the TPVS was low pressure and showed a smooth, regular pattern corresponding to respiration. This waveform was reliable for verifying the needle tip in the TPVS. Hence, these findings can be applied as an adjunct technique to perform TPVB combined with an anatomical-based method or an UG-TPVB, especially when the needle cannot be seen clearly.

## Data Availability

Figshare: data analysis figshare new.xls, https://doi.org/10.6084/m9.figshare.24189234.v2. ^30^ This project contains the following underlying data:
- Data analysis figshare.xls Data are available under the terms of the Creative Commons Attribution 4.0 International license (CC-BY 4.0).
