# Supplementary material for: Waveform characteristics in thoracic paravertebral space: a prospective observational study
Source: F1000Res. 2025 Jan 27;13:150. Originally published 2024 Mar 1. [Version 2] doi: 10.12688/f1000research.139904.2 (PMC11807241; doi:10.12688/f1000research.139904.2)
Supplement: Peer review history

# Reviewer report: Reviewer response for version 2

**DOI:** 10.5256/f1000research.177025.r362659

**Published:** 2025-02-08

**Authors:** Sirirat Tribuddharat

**Affiliations:** 1 Department of Anesthesiology, Faculty of Medicine, Khon Kaen University, Khon Kaen, Thailand

All comments have been addressed, and the manuscript is now ready for indexing.

Is the work clearly and accurately presented and does it cite the current literature?

Yes

If applicable, is the statistical analysis and its interpretation appropriate?

Partly

Are all the source data underlying the results available to ensure full reproducibility?

No

Is the study design appropriate and is the work technically sound?

Partly

Are the conclusions drawn adequately supported by the results?

Yes

Are sufficient details of methods and analysis provided to allow replication by others?

Partly

Reviewer Expertise:

Anesthesia; Cardiac anesthesia; regional anesthesia; hemodynamics monitoring

I confirm that I have read this submission and believe that I have an appropriate level of expertise to confirm that it is of an acceptable scientific standard.

# Reviewer report: Reviewer response for version 1

**DOI:** 10.5256/f1000research.153219.r345406

**Published:** 2024-12-12

**Authors:** Sirirat Tribuddharat

**Affiliations:** 1 Department of Anesthesiology, Faculty of Medicine, Khon Kaen University, Khon Kaen, Thailand

This study presents a novel method for identifying the thoracic paravertebral space, offering an alternative to ultrasound guidance. While well-designed, some areas require further clarification:

1. Numerals: Avoid beginning sentences with numerals. "50" and "98" in the Abstract should be written as "Fifty" and "Ninety-eight," respectively, and similar changes should be made throughout the manuscript.
2. Abstract Results: "Chronic pleural inflammation" should be removed from the Abstract Results, as it's not addressed in the manuscript's Results section.
3. Introduction P-values: Include p-values for the percentages "(94% and 72%, respectively)" and "(13.8% and 22.2%, respectively)" presented in the Introduction.
4. Complication Order: Reorder the listed complications—hypotension (4.6%), vascular puncture (3.8%), pleural puncture (1.1%), pneumothorax (0.5%), hematoma (1.9%), and epidural/spinal spread (1.1%)—according to their incidence.
5. Methods Wording: In the Methods section, change "provided by the Ethics Committee" to "approved by the Ethics Committee" and "obtained preoperative information and provided informed consent" to "provided preoperative information and obtained informed consent."
6. Sample Size Rationale: Justify the sample size of 50 patients, potentially with a power analysis or reference to similar studies.
7. ASA Definition Removal: Remove the detailed ASA physical status definitions, as referencing #16 is sufficient.
8. Ventilation Clarification: Address the discrepancy between the stated inspiratory pressure (20 mmHg) and the mean inspiratory pressure reported in Table 1 (14 ± 1.54 mmHg). Include details on tidal volume and EtCO2 adjustments.
9. "A" Waveform Definition: Remove "a mean value of ≤25 mmHg" from the "A" waveform definition, as it has mean values with a range between 3.0-47.0 or 8.0-26.0 (Table 2). Please clarify.
10. Unsuccessful Block Definition: Delete "or an NRS score at rest ≥3" and all other mentions of NRS scores, as they are not relevant per reference #3. I suggest deleting ‘Numeric Rating Scale (NRS) scores’ because they were not presented in Table 1.
11. Table 1 Formatting: Remove ", mean±SD" and ", n (%)" from Table 1 contents and "(range)" from its caption. Change the caption to "Data are presented as mean ± SD or n (%)".
12. Figure 2 Label: Correct the "ICP" label in Figure 2. Provide the correct term (e.g., "Intrathoracic Pressure").
13. Redo-Thoracotomy Count: Reconcile the inconsistent reporting of redo-thoracotomy patients (5 in Results, 6 in Discussion, 4 in Table 2).
14. Table 2 P-values: Add p-values for intramuscular pressure, SCTL pressure, TPVS pressure, and respiratory waveform presence for redo and non-redo thoracotomy groups at T4/5 and T6/7.
15. Table 2 Formatting: Change the Table 2 caption to "Data are presented as median (range) or n (%)". Remove ", %" from the first column. Present "Respiratory waveform present" values as n (%) and change all other percentage values (e.g., 94.0, 100.0, 93.5) accordingly.
16. Table 3 Discrepancy: Resolve the discrepancy between the 98 (98%) regular sine waveforms in Table 3 and the 94.0% respiratory waveform presence in Table 2.
17. Table 3 Captions: Remove "Sensitivity, Specificity, ROC area, Positive predictive value, and Negative predictive value" captions from Table 3; these belong in Table 4.
18. NRS Score Paragraph Removal: Delete the paragraph discussing NRS scores, as it's not relevant to the study's objective.
19. In the Discussion, the authors stated that ‘The study showed that 96% of the subjects had similar respiratory-like pattern in the TPVS’, where did 96% come from? According to Table 3, this number should be 98%. Please clarify.
20. To assess the efficacy of sine waveform visualization for identifying the thoracic paravertebral space (TPVS), comparison with a reference standard is essential. While not a true gold standard, ultrasound-guided TPVB (UG-TPVB) is the de facto standard for TPVS identification in clinical practice. Therefore, the sine waveform's visibility should be compared directly with UG-TPVB confirmation of needle tip location within the TPVS (e.g., % sine waveform visibility vs. US-TPVB confirmation). Comparing sine wave visibility with block success (Table 4) is inappropriate. The current criterion for unsuccessful block—inability to confirm more than three levels of dermatomal blockade—introduces numerous confounding factors unrelated to TPVS identification. Block success itself is not a valid reference standard for accurate needle placement within the TPVS as confirmed by US-TPVB.
21. Table 4 Revision: Recalculate and revise Table 4 to compare sine waveform visibility directly with US-TPVB confirmation, using a total of 100 tests.
22. Discussion Wording: Change "Eleventh" to "Eleven" in the Discussion.

Is the work clearly and accurately presented and does it cite the current literature?

Yes

If applicable, is the statistical analysis and its interpretation appropriate?

Partly

Are all the source data underlying the results available to ensure full reproducibility?

No

Is the study design appropriate and is the work technically sound?

Partly

Are the conclusions drawn adequately supported by the results?

Yes

Are sufficient details of methods and analysis provided to allow replication by others?

Partly

Reviewer Expertise:

Anesthesia; Cardiac anesthesia; regional anesthesia; hemodynamics monitoring

I confirm that I have read this submission and believe that I have an appropriate level of expertise to confirm that it is of an acceptable scientific standard, however I have significant reservations, as outlined above.

# Response

**Published:** 2025-01-14

**Authors:** Amorn Vijitpavan

**Affiliations:** Anesthesiology (Ramathibodi), Mahidol University, Ratchathewi, Bangkok, Thailand

**Respond to the reviewer questions**

**Question 1**: Numerals: Avoid beginning sentences with numerals. "50" and "98" in the Abstract should be written as "Fifty" and "Ninety-eight," respectively, and similar changes should be made throughout the manuscript.

**Answer 1**: This issue has been revised as per the editor's suggestions, in accordance with the journal format, as shown in the attached document. The journal editor suggests using numbers rather than letters.

**Question 2**: Abstract Results: "Chronic pleural inflammation" should be removed from the Abstract Results, as it's not addressed in the manuscript's Results section.

**Answer 2**: We have removed the term “chronic pleural inflammation” from the abstract results.

**Question 3**: Introduction P-values: Include p-values for the percentages "(94% and 72%, respectively)" and "(13.8% and 22.2%, respectively)" presented in the Introduction.

**Answer 3**: We added the p-values to (94% and 72%, p = 0.024, respectively) and (13.8% and 22.2%, p = 0.54, respectively), as suggested.

**Question 4**: Complication Order: Reorder the listed complications—hypotension (4.6%), vascular puncture (3.8%), pleural puncture (1.1%), pneumothorax (0.5%), hematoma (1.9%), and epidural/spinal spread (1.1%)—according to their incidence.

**Answer 4**: We have reordered the words by occurrence, from most to least, as suggested, as follows: hypotension (4.6%), vascular puncture (3.8%), hematoma (1.9%), pleural puncture(1.1%), epidural or spinal spread (1.1%), and pneumothorax (0.5%).

**Question 5**: Methods Wording: In the Methods section, change "provided by the Ethics Committee" to "approved by the Ethics Committee" and "obtained preoperative information and provided informed consent" to "provided preoperative information and obtained informed consent."

**Answer 5**: I have rearranged the sentence as suggested, as follows: “approved by the Ethics Committee” and “provided preoperative information and obtained informed consent”, as suggested.

**Question 6**: Sample Size Rationale: Justify the sample size of 50 patients, potentially with a power analysis or reference to similar studies.

**Answer 6**: This is an observational study of pressure levels and waveform characteristics in the paravertebral space, which has never been studied before. The sample size for this study was derived from the study period, during which participants were observed from February 9, 2021, to May 17, 2021—approximately 3 months. A total of 50 cases were included.

**Question 7**: ASA Definition Removal: Remove the detailed ASA physical status definitions, as referencing #16 is sufficient.

**Answer 7**: Thank you for your helpful suggestion. Since the editor of the journal recommended adding a definition of ASA physical status in the previous review, we will retain the definition in the manuscript.

**Question 8**: Ventilation Clarification: Address the discrepancy between the stated inspiratory pressure (20 mmHg) and the mean inspiratory pressure reported in Table 1 (14 ± 1.54 mmHg). Include details on tidal volume and EtCO2 adjustments.

**Answer 8**: Because the thoracic paravertebral space is adjacent to the thoracic cavity, the researchers concerned that the pressure generated by positive pressure ventilation might influence the pressure and waveform characteristics being studied. Therefore, the same PCV ventilation settings (20 mmHg) were applied to all cases during the brief study period. The values reported in Table 1 represent the actual mean airway pressures (14 ± 1.54 mmHg), which typically do not match the set pressure (20 mmHg) in the PCV mode. We aim to demonstrate that both the set pressure level and the mean airway pressure were not significantly different across participants. Adjustments to tidal volume and EtCO2 could affect the ventilated pressure; however, since the study duration was brief, the varation of EtCO2 should not harm the participants.

**Question 9**: "A" Waveform Definition: Remove "a mean value of ≤25 mmHg" from the "A" waveform definition, as it has mean values with a range between 3.0-47.0 or 8.0-26.0 (Table 2). Please clarify.

**Answer 9**: This study examines the pressure in the paravertebral space and the respiratory-like pattern of waveforms that may be observed in this space. The definitions of waveforms A, B, and C combine both pressure and waveform characteristics, as follows: Waveform A is a smooth, regular sine wave resembling the respiratory pattern, with mean pressure in the space ≤ 25 mmHg; Waveform B is an irregular, coarse, wavy line with pressure < 40 mmHg; and Waveform C is a tense, straight line with pressure > 40 mmHg. Table 2 shows the pressure and waveform data, with the numbers (3, 47.0) or (8.0, 26.0) representing the median values measured in the paravertebral space. As noted by the reviewer, pressures over 25 mmHg, such as those corresponding to (3, 47.0) or (8.0, 26.0), were classified as either waveform C or B, not waveform A. Only two patients were found to have pressure greater than 25 mmHg in the thoracic paravertebral space.

 To clarify, we have provided additional information about the waveform characteristics in the Methods section, as shown in Table 3. The “A” waveform, or typical waveform, was defined as a smooth and regular sine wave resembling the respiratory pattern, with a mean pressure ≤ 25 mmHg. The “B” waveform was defined as an irregular, coarse, wavy line with a mean pressure ≤ 40 mmHg. The “C” waveform was defined by a tense, straight line with a mean pressure > 40 mmHg.

**Question 10**: Unsuccessful Block Definition: Delete "or an NRS score at rest ≥3" and all other mentions of NRS scores, as they are not relevant per reference #3. I suggest deleting ‘Numeric Rating Scale (NRS) scores’ because they were not presented in Table 1.

**Answer 10**: We agree to remove the NRS (Numeric Rating Scale) scores from the section on the definition of unsuccessful blockade and from all other parts of the manuscript where NRS is mentioned.

**Question 11**: Table 1 Formatting: Remove ", mean±SD" and ", n (%)" from Table 1 contents and "(range)" from its caption. Change the caption to "Data are presented as mean ± SD or n (%)".

**Answer 11**: We removed the words “mean±SD” and “n” from Table 1 and the word “range” from the caption, and changed the caption to “Data are presented as mean±SD or n(%)” as recommended.

**Question 12**: Figure 2 Label: Correct the "ICP" label in Figure 2. Provide the correct term (e.g., "Intrathoracic Pressure").

**Answer 12**: We agreed to omit parts of the ICP text from Figure 2 to prevent confusion among readers who might mistake it for intracranial pressure, as this study focuses on the paravertebral space.

**Question 13**: Redo-Thoracotomy Count: Reconcile the inconsistent reporting of redo-thoracotomy patients (5 in Results, 6 in Discussion, 4 in Table 2).

**Answer 13**: Thank you for your help in checking. We would like to change the number of participants who underwent redo-thoracotomy from 5 patients to 4 patients in the results section. The number of 6 patients in the discussion section is correct because it includes 4 redo-thoracotomy cases and 2 empyema cases. Both the redo-thoracotomy and empyema cases involve issues with pleural integrity, which is why we reported them together.

**Question 14**: Table 2 P-values: Add p-values for intramuscular pressure, SCTL pressure, TPVS pressure, and respiratory waveform presence for redo and non-redo thoracotomy groups at T4/5 and T6/7.

**Answer 14**: I have added the p-values to Table 2 for intramuscular pressure, SCTL, TPVS pressure, and the presence of respiratory waveforms for the Redo and non-redo thoracotomy groups at the T4/5 and T6/7 levels.

**Question 15**: Table 2 Formatting: Change the Table 2 caption to "Data are presented as median (range) or n (%)". Remove ", %" from the first column. Present "Respiratory waveform present" values as n (%) and change all other percentage values (e.g., 94.0, 100.0, 93.5) accordingly.

**Answer 15**: As per your recommendation. We have adjusted Table 2 as follows:

 1. In the table header, we removed the word “Mean” in the “Mean pressure values” and added “(n=100)”  for clarity.

 2. In columns 2 and 3, specifying T4/5 and T5/6, we removed the word “median (range)” replaced it with “(n=50)”.

 3. We removed the “%”  at the end of “Respiratory waveform present, %” and presented it as n(%) instead.

 4. We recalculated the data, correcting the value from 93.5 to 95.6.

 5. The caption of Table 2 was edited to  “Data are presented as median (range) or n(%)”.

**Question 16**: Table 3 Discrepancy: Resolve the discrepancy between the 98 (98%) regular sine waveforms in Table 3 and the 94.0% respiratory waveform presence in Table 2.

**Answer 16**: The number 94 in Table 2 represents the number of times the respiratory waveform occurred in the observational study, presented as percentages. It is not specified whether this occurred in the thoracic paravertebral space. In this study, only one case was found to have a respiratory waveform in the SCTL (superior costotransverse ligament) position, which was also observed in the thoracic paravertebral space. Additionally, two cases did not exhibit the respiratory-like waveform in the paravertebral space. Therefore, in Table 2, the number 94 has been revised to 96 (48/50 at T4/5 for all participants), and the number 93.5 has been revised to 95.6 (44/46 in the not-redo-thoracotomy group), with these numbers presented as percentages. The number 98 in Table 3 represents the total number of events in which the respiratory waveform occurred in the paravertebral space (two levels of paravertebral block for one patient).

**Question 17**: Table 3 Captions: Remove "Sensitivity, Specificity, ROC area, Positive predictive value, and Negative predictive value" captions from Table 3; these belong in Table 4.

**Answer 17**: In Table 3, we reported the use of ultrasound as the gold standard for identifying the paravertebral space before measuring the pressure and waveform characteristics. Therefore, we would like to retain these data. Additionally, we used the success of the blockade to reconfirm the paravertebral space after the blockade, as shown in Table 4.

**Question 18**: NRS Score Paragraph Removal: Delete the paragraph discussing NRS scores, as it's not relevant to the study's objective.

**Answer 18**: We have removed the paragraph discussing the NRS, as suggested.

**Question 19**: In the Discussion, the authors stated that ‘The study showed that 96% of the subjects had similar respiratory-like pattern in the TPVS’, where did 96% come from? According to Table 3, this number should be 98%. Please clarify.

**Answer 19**: As answered in question 16, the number 96% comes from the events showing the respiratory-like waveform, which includes 48 cases out of 50. However, the number 98 represents the total events showing the respiratory-like waveform from 100 events involving fifty patients.

**Question 20**: To assess the efficacy of sine waveform visualization for identifying the thoracic paravertebral space (TPVS), comparison with a reference standard is essential. While not a true gold standard, ultrasound-guided TPVB (UG-TPVB) is the de facto standard for TPVS identification in clinical practice. Therefore, the sine waveform's visibility should be compared directly with UG-TPVB confirmation of needle tip location within the TPVS (e.g., % sine waveform visibility vs. US-TPVB confirmation). Comparing sine wave visibility with block success (Table 4) is inappropriate. The current criterion for unsuccessful block—inability to confirm more than three levels of dermatomal blockade—introduces numerous confounding factors unrelated to TPVS identification. Block success itself is not a valid reference standard for accurate needle placement within the TPVS as confirmed by US-TPVB.

**Answer 20**: This is because the researcher needed to understand the pressure and waveform characteristics in the paravertebral space, as these have never been studied before. The researcher had previously studied the characteristics of waves found in the epidural space and discovered that they resembled arterial waves, which were used to determine the location of the epidural space. In the paravertebral space, which is connected to the epidural space and adjacent to the thoracic cavity, there may be arterial waves or possibly waves similar to respiratory waves following breathing. Therefore, the researcher aimed to study the nature of the waves in the paravertebral space, their form, and whether they could be used to determine the location of the paravertebral space. In this study, ultrasound was used to identify the location of the paravertebral space. However, even with ultrasound, the tip of the needle could sometimes not be clearly visualized, and it was necessary to confirm that the location identified with ultrasound was indeed the paravertebral space. Therefore, the researcher also tested the level of analgesia after the blockade. In future studies, the use of respiratory waveforms may be compared with ultrasound for determining the location of the paravertebral space.

**Question 21**: Table 4 Revision: Recalculate and revise Table 4 to compare sine waveform visibility directly with US-TPVB confirmation, using a total of 100 tests.

**Answer 21**: Table 4 presents the relationship between the visibility of the respiratory waveform and the success of the block, as determined by testing the level of analgesia. The study involved 50 patients, and the level of analgesia was tested after they had recovered from anesthesia. Therefore, the calculations were based on 50 patients, not 100. We acknowledge that this is a limitation of the study, as the analgesic level should have been tested before the patient fell asleep for each blockade. We addressed this point in the limitation section.

**Question 22**: Discussion Wording: Change "Eleventh" to "Eleven" in the Discussion.

**Answer 22**: An error occurred. We have changed the word “eleventh” to “eleven”.

# Reviewer report: Reviewer response for version 1

**DOI:** 10.5256/f1000research.153219.r319560

**Published:** 2024-09-13

**Authors:** Sasikaan Nimmaanrat

**Affiliations:** 1 Songklanagarind Hospital, Faculty of Medicine, Prince of Songkla University, Hat Yai, Thailand

This manuscript is well written.

 The research question was to answer the knowledge gap of TPVS waveform patterns in patients with "controlled ventilation" under general anesthesia.

 The methodology was well designed. The results were clearly presented. 

 The discussion was adequate on similarities and dissimilarities with other publications with logic explanations.

Is the work clearly and accurately presented and does it cite the current literature?

Yes

If applicable, is the statistical analysis and its interpretation appropriate?

Yes

Are all the source data underlying the results available to ensure full reproducibility?

Yes

Is the study design appropriate and is the work technically sound?

Yes

Are the conclusions drawn adequately supported by the results?

Yes

Are sufficient details of methods and analysis provided to allow replication by others?

Yes

Reviewer Expertise:

Pain management, Anesthesia, Quality Assurance

I confirm that I have read this submission and believe that I have an appropriate level of expertise to confirm that it is of an acceptable scientific standard.
